# Spatial representability of neuronal activity

**DOI:** 10.1038/s41598-021-00281-y

**Published:** 2021-10-25

**Authors:** D. Akhtiamov, A. G. Cohn, Y. Dabaghian

**Affiliations:** 1 Einstein institute of Mathematics, The Hebrew University, Jerusalem, 9190401 Israel; 2 School of Computing, University of Leeds, Woodhouse Lane, Leeds, LS9 2JT UK; 3 Luzhong Institute of Safety, Environmental Protection Engineering and Materials, Qingdao University of Science & Technology, Zibo, 255000 China; 4 School of Mechanical and Electrical Engineering, Qingdao University of Science and Technology, Qingdao, 260061 China; 5 Department of Computer Science and Technology, Tongji University, Shanghai, 211985 China; 6 School of Civil Engineering, Shandong University, Jinan, 250061 China; 7 Department of Neurology, The University of Texas McGovern Medical School, 6431 Fannin St, Houston, TX 77030 USA

**Keywords:** Learning algorithms, Neural decoding, Neural encoding

## Abstract

A common approach to interpreting spiking activity is based on identifying the firing fields—regions in physical or configuration spaces that elicit responses of neurons. Common examples include hippocampal place cells that fire at preferred locations in the navigated environment, head direction cells that fire at preferred orientations of the animal’s head, view cells that respond to preferred spots in the visual field, etc. In all these cases, firing fields were discovered empirically, by trial and error. We argue that the existence and a number of properties of the firing fields can be established theoretically, through topological analyses of the neuronal spiking activity. In particular, we use Leray criterion powered by persistent homology theory, Eckhoff conditions and Region Connection Calculus to verify consistency of neuronal responses with a single coherent representation of space.

## Introduction

**Figure 1:**
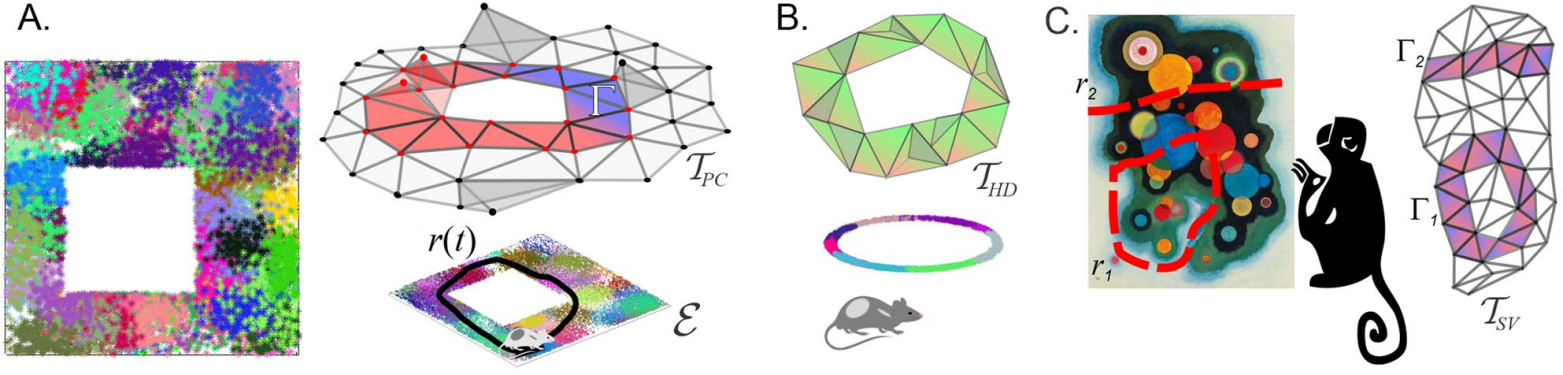
Spatial maps. (**A**) A simulated place field map of a small ($1m\times 1m$) environment ${\mathcal {E}}$, similar to the arenas used in typical electrophysiological experiments^66,67^. Dots represent spikes produced by the individual cells (color-coded); their locations mark the rat’s position at the time of spiking. The pool of place cell coactivities is schematically represented by a coactivity complex ${\mathcal {T}}_{PC}$ (top right). The navigated trajectory *r*(*t*) induces a sequence of activated simplexes—a simplicial path $\Gamma \in {\mathcal {T}}_{PC}$. (**B**) The head direction cell combinations ignited during navigation induce a coactivity complex ${\mathcal {T}}_{HD}$ (top). The corresponding head direction fields cover a unit circle—the space of directions (bottom). (**C**) Spatial view cells activate when the primate gazes at their respective preferred domains in the visual field (left). The curves $r_1(t)$ and $r_2(t)$ traced by the monkey’s gaze induce simplicial paths $\Gamma _1$ and $\Gamma _2$ running through the corresponding coactivity complex ${\mathcal {T}}_{VC}$ (right).

Physiological mechanisms underlying the brain’s ability to process spatial information are discovered by relating parameters of neuronal spiking with characteristics of the external world. In many cases, it is possible to link neuronal activity to geometric or topological aspects of a certain space—either physical or auxiliary. For example, a key insight into neuronal computations implemented by the mammalian hippocampus is due to O’Keefe and Dostrovsky’s discovery of a correlation between the firing rate of principal neurons in rodents’ hippocampi and the animals’ spatial location^1–3^. This discovery allowed interpreting these neurons’ spiking activity, henceforth called *place cells*, as representations of spatial domains—their respective *place fields* (Fig. 1A^4^) (Throughout the text, terminological definitions are given in *italics*.). It then became possible to use place field layout in the navigated environment ${\mathcal {E}}$—the *place field map* $M_{{\mathcal {E}}}$—to decode the animal’s ongoing location^5–8^, and even to interpret the place cells’ off-line activity during quiescent stages of behavior or in sleep^9–14^, which define our current understanding of the hippocampus’ contribution to spatial awareness^15–18^.

In the 90s, a similar line of arguments was applied to cells discovered in rat’s postsubiculum and in other parts of the brain^19–21^, which fire at a particular orientation of the animal’s head. The angular domains where such *head direction cells* become active can be viewed as one-dimensional (1*D*) *head direction fields* in the circular space of planar directions, $S^1$—in direct analogy with the hippocampal place fields in the navigated space (Fig. 1B). The corresponding * head direction map*, $M_{S^1}$, defines the order in which the head direction cells spike during the rat’s movements and the role of these cells in spatial orientation^20–22^. Recently, place cells and head directions cells were discovered in bats’ hippocampi; in contrast with rodents who navigate two-dimensional (2*D*) surfaces (see however^23–26^), bat’s voluminous place fields cover three-dimensional (3*D*) environments and their head direction fields cover 2*D* tori^27,28^.

The *spatial view cells*, discovered in the late 90s, activate when a primate is looking at their preferred spots in the environment (Fig. 1C), regardless of the head direction or location^29–31^. Correlating these cells’ spike timing with the positions of the *view fields* helped understanding mechanisms of storing and retrieving episodic memories, remembering object locations, etc.^32,33^. The principles of information processing in sensory and somatosensory cortices were also deciphered in terms of receptive fields—domains in sensory spaces, whose stimulation elicits in spiking responses of the corresponding neurons^34–39^.

In all these cases, referencing an individual neuron’s activity to a particular domain in a suitable *representing space* *X*^40^ is key for understanding its contribution and for reasoning about functions of neuronal ensembles in terms of the corresponding “maps”^16–18^. This raises a natural question: when is a “spatial” interpretation of neuronal activity at all possible, i.e., when there might exist a correspondence between the patterns of neuronal activity and regions in low-dimensional space?

## Approach

### A mathematical perspective

On this question is suggested by the simplicial topology framework^41,42^. Specifically, if a combination of coactive cells, $c_{i_0},c_{i_1},\ldots ,c_{i_k}$ is represented by an abstract *coactivity simplex* (for definitions see “Methods” section)

1 $$\begin{aligned} \sigma _i = [c_{i_0},c_{i_1},\ldots ,c_{i_k}], \end{aligned}$$

then the net pool of coactivities observed by the time *t* forms a simplicial complex

2 $$\begin{aligned} {\mathcal {T}}(t)=\cup _{i}\sigma _i. \end{aligned}$$

On the other hand^42–45^, a similar construction can be carried out for a space *X* covered by a set of regions $\upsilon _i$,

3 $$\begin{aligned} X=\cup _i\upsilon _i. \end{aligned}$$

If each nonempty overlap between these regions,

4 $$\begin{aligned} \upsilon _{\sigma _i}\equiv \upsilon _{i_0}\cap \upsilon _{i_1}\cap \ldots \cap \upsilon _{i_k}\ne \varnothing , \end{aligned}$$

is formally represented by an abstract simplex,

5 $$\begin{aligned} \nu _{\sigma _i}=[\upsilon _{i_0},\upsilon _{i_1},\ldots ,\upsilon _{i_k}], \end{aligned}$$

then the cover (3) generates another simplicial complex, known as its *Čech* or *nerve* complex

6 $$\begin{aligned} {\mathcal {N}}_X=\cup _i\nu _{\sigma _i}, \end{aligned}$$

which is a spatial analogue of the coactivity complex (2). The idea is hence the following: if there is a correspondence between neurons’ spiking and spatial regions, then multi-cell coactivities can be viewed as representations of their firing fields’ overlaps^46–48^. Thus, the question whether a given pool of neuronal activity corresponds to a spatial map can be answered by verifying *representability* of the corresponding coactivity complex ${\mathcal {T}}(t)$, i.e., testing whether the latter has a structure of a nerve ${\mathcal {N}}_X$ of some cover in a low-dimensional representing space *X*.

### Implementation

As it turns out, representable simplicial complexes exhibit several characteristic properties that distinguish them among generic simplicial complexes^49,50^. Verifying these properties over biologically relevant 1*D*, 2*D* and 3*D* representing spaces is a tractable problem^49,51^, although exact algorithms for performing such a verification are not known—only in 1*D* are some methods available^53–57^. Nevertheless, there exist explicit criteria that allow limiting the dimensionality of the representing space *X* and eliminating manifestly non-representable complexes based on their homologies, combinatorics of simplexes and other intrinsic topological properties, which will be used below.

Specifically, according to the *Leray criterion*, a complex $\Sigma$ representable in *D* dimensions should not contain non-contractible gaps, cavities or other topological defects in dimensionalities higher than $(D-1)$^58^. Formally, it is required that the homological groups of $\Sigma$ and hence its Betti numbers should vanish in these dimensions, $b_{i\ge D}(\Sigma )=0$. Moreover, the Betti numbers of all the subcomplexes $\Sigma _x$ of $\Sigma$, induced by a fraction *x* of its vertexes should also vanish, $b_{i\ge D}(\Sigma _x)=0$. In the case of coactivity complexes, such subcomplexes ${\mathcal {T}}_x \subseteq {\mathcal {T}}$ have a particularly transparent interpretation: they are the ones generated by $x\%$ of the active cells. According to the second criterion, the number of simplexes in all dimensions of $\Sigma$ must obey *Eckhoff’s inequalities*—a set of combinatorial relationships discussed in^59–62^ and listed in the Methods” section, where we also briefly detail the Leray criterion^49,58,62–64^.

Previous topological studies of the coactivity data were motivated by the Alexandrov-Čech theorem^42–45^, which posits that the homologies of the nerve complexes produced by the “good” covers (i.e., the ones with contractible overlaps (4), see^65^), should match the homologies of the underlying space *X*, $H_{*}({\mathcal {N}})= H_{*}(X)$, i.e., have the same number of pieces, holes, tunnels, etc. Specifically, this construction was applied to the place cell coactivity complexes, whose representability was presumed^46–48^. Persistent homology theory^68–73^ was used to trace the dynamics of the Betti numbers $b_{i \le D}({\mathcal {T}})$ in physical dimensionalities $D\le 2$^74–79^ and $D\le 3$^79,80^, to detect whether and when these numbers match the Betti numbers of the environment, $b_{i\le D}({\mathcal {E}})$, and how this dynamics depends on spiking parameters. It was demonstrated, e.g., that for a wide range of the firing rates and place field sizes referred to as the *Learning Region*, ${\mathcal {L}}({\mathcal {E}})$, the low-dimensional Betti numbers of ${\mathcal {T}}(t)$ converge to their physical values after a certain period $T_{\min }$, neurobiologically interpreted as the minimal time required to “learn” the topology of the environment ($b_{i\le D}({\mathcal {T}}(t))= b_{i\le D} ({\mathcal {E}})$, $t\ge T_{\min }$^48^).

Moreover, it became possible to asses the contribution of various physiological parameters—from brain waves to synapses—to producing and sustaining the topological shape of ${\mathcal {T}}$^74–80^. In addition, the coactivity complexes were used for contextualizing the ongoing spiking activity and linking its structure to the animal’s behavior. For example, it was shown that a trajectory $\gamma (t)$ tracing through a sequence of firing domains $\upsilon _{\sigma _i}$, produces a “simplicial path” $\Gamma$—a succession of active simplexes that captures the shape of $\gamma (t)$ and allows interpreting the animal’s active behavior^5–8^ and its “off-line” memory explorations^9–14,20,32^ (Fig. 1).

Together, these arguments suggest that experimentally discovered representing spaces and firing fields serve as explicit models of the cognitive maps emerging from neuronal activity—a perspective that is currently widely accepted in neuroscience. However, this view requires verification, since the empirically identified firing fields may be contextual offshoots or projections from some higher-dimensional constructs—in the words of H. Eichenbaum, “*hippocampal representations are maps of cognition, not maps of physical space*”^81^. The way of addressing this question is straightforward: if the spiking activity is intrinsically spatial, i.e., if neurons represent spatial domains, then the coactivity complexes generated by the corresponding neuronal ensembles should be representable—an explicit property that can be confirmed or refuted using Leray, Eckhoff and other criteria. In the following, we apply these criteria to several types of neuronal activity, both simulated and experimentally recorded, and discuss the results.

## Results

### Simplicial topology approach

The conventional theory of representability addresses properties of “static” simplicial complexes^49–65^. In contrast, the coactivity complexes are dynamic structures that can be viewed as time-ordered agglomerates of simplexes, restructuring at the moments $t_1<t_2<t_3\ldots$,

7 $$\begin{aligned} {\mathcal {T}}(t_1)\subseteq {\mathcal {T}}(t_2)\subseteq {\mathcal {T}}(t_3)\ldots \,. \end{aligned}$$

The exact organization of each complex in the sequence (7) depends on the specifics of the underlying spiking activity, e.g., the initial state of the network, its subsequent dynamics, spiking mechanisms and so forth (in case of the place fields, think of the starting point of navigation, shape of the trajectory, speed, etc.). Thus, verifying representability of these complexes requires testing whether Eckhoff, Leray and other criteria are valid at each moment *t*.

We constructed coactivity complexes by simulating the rat’s navigation through a planar environment ${\mathcal {E}}$ commonly used in electrophysiological experiments (Fig. 1A, see also^66,67^). The neuronal spikings in this case are generated as responses to the rat’s appearances within preconstructed, convex firing domains, e.g., stepping into randomly scattered place fields or facing towards head direction fields centered around randomly chosen preferred angles (see Methods” section and Methods in^48,74,82^). While the resulting nerve complexes (6) are 2*D*-representable by design, we inquired whether the corresponding coactivity complexes are also representable, i.e., whether the activity of individual neurons intrinsically represents regions and whether connectivity between these regions is similar to the connectivity between the underlying auxiliary firing fields.

**Figure 2:**
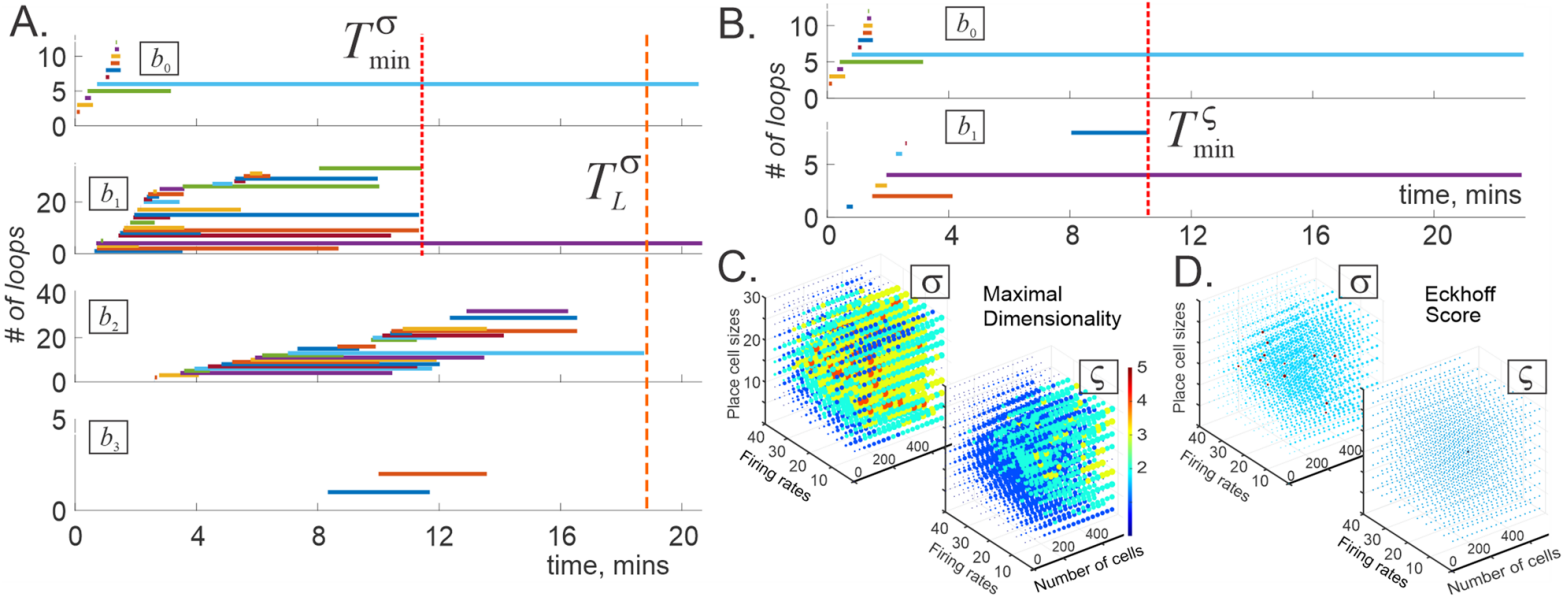
Persistent Leray dimension. (**A**) The Leray dimensionality of the coincidence-detector complex ${\mathcal {T}}_{\sigma }(t)$ constructed for an ensemble of $N_c=300$ place cells can rise to $D({\mathcal {T}}_{\sigma })=4$ (here the mean maximal firing rate is $f=12$ Hz, mean place field size $s=22$ cm; environment ${\mathcal {E}}$ same as on Fig. 1A). In about 17 min—the corresponding Leray period $T_L$—the dimensionality drops to $D({\mathcal {T}}_{\sigma })={\bar{D}}_L({\mathcal {T}}_{\sigma })=1$, after which the spiking patterns can be intrinsically interpreted in terms of planar firing fields. Note that the Leray period in this case is longer than the minimal learning time evaluated based on the lower-dimensional Betti numbers $b_{0,1}({\mathcal {T}}_{\sigma })$, $T_L > T_{\min }^{\sigma }$. Shown are all the non-zero Betti numbers of ${\mathcal {T}}_{\sigma }(t)$. (**B**) Timelines of the topological loops in a spike-integrating coactivity complex, evaluated for the same cell population in the same environment yields the persistent Leray dimensionality ${\bar{D}}_L({\mathcal {T}}_{\varsigma })=1$ from the onset. The disappearance of spurious 0*D* loops in about 11 minutes marks the end of the learning period $T_{\min }^{\varsigma }(t)$. Note that the number of spurious loops in ${\mathcal {T}}_{\varsigma }(t)$ is significantly lower than in ${\mathcal {T}}_{\sigma }(t)$. (**C**) Maximal dimensionality of the topological loops in ${\mathcal {T}}_{\sigma ,\varsigma }(t)$. (**D**) The Eckhoff conditions are satisfied for nearly all coincidence-detecting complexes ${\mathcal {T}}_{\sigma }(t)$ (left panel, occasional exceptions are shown by red dots) and for all spike integrating complexes ${\mathcal {T}}_{\varsigma }(t)$ (right panel).

Simulations show that *persistent Leray dimensionality* ${\bar{D}}_L$ (above which the spurious loops in ${\mathcal {T}}(t)$ vanish, ${\bar{D}}_L=\min (\{D:b_{i>D}({\mathcal {T}}(t))=0,\,t\ge T_L\})$, see also^83^) eventually settles at ${\bar{D}}_L =1$ for most complexes, implying that neuronal activity defines a proper planar map. However, this mapping requires time—a *Leray period* $T_L$—which, for the maps populating the learning region ${\mathcal {L}}({\mathcal {E}})$, is typically similar to the learning time $T_{\min }$ (Fig. 2A,B).

Whether a particular value of $T_L$ is shorter or longer than the corresponding $T_{\min }$ depends on how exactly the coactivity complex is constructed, e.g., whether the simplexes (1) correspond to simultaneously igniting cells groups or assembled from lower-order combinations over an extended period $\varpi$^84^. Physiologically, the former corresponds to the case when spiking outputs are processed by “coincidence-detector” neurons in the downstream networks and the latter to the case when lower-order coactivities are collected over a certain “spike integration window” $\varpi$—longer than the simultaneity detection timescale *w*^85–87^. Different readout neurons or networks may have different integration periods; to simplify the model, we started by extending the parameter $\varpi$ to the entire navigation period for all cells and cell groups.

The lowest order of coactivity involves spiking cell pairs^88^, which together define a coactivity graph ${\mathcal {G}}$^89,90^. The cliques $\varsigma$ of this graph produce a *clique coactivity complex* ${\mathcal {T}}_{\varsigma }$ that generalizes the *simplicial coactivity complex* ${\mathcal {T}}_{\sigma }$, built from simultaneously detected simplexes^75–80^. As it turns out, the “coincidence detecting” and the “spike integrating” complexes have different topological dynamics: the former are more likely to start off with a higher Leray dimensionality, ${\bar{D}}_L\ge 3$, that then reduces to ${\bar{D}}_L\le 2$ (Fig. 2A), whereas the latter tend to be more stable, lower-dimensional and have shorter Leray and learning times (Fig. 2B).

To test the induced subcomplexes of each ${\mathcal {T}}$, we selected random subcollections of cells containing $x=50\%$, $x=33\%$, $x=25\%$ and $x=20\%$ of the original neuronal ensemble, and found that if the original complex ${\mathcal {T}}\equiv {\mathcal {T}}_{x=1}$ is representable, then its subcomplexes, ${\mathcal {T}}_{x<1}\subseteq {\mathcal {T}}$, typically require less time to pass the Leray criterion, $T_L({\mathcal {T}}_{x<1})\le T_L({\mathcal {T}})$, and that for $x>50\%$ the Leray times saturate, $T_L({\mathcal {T}}_{x>0.5})\approx T_L({\mathcal {T}})$. Thus, the Leray time of the full complex, $T_L({\mathcal {T}})$, can be used as a general estimate of the timescale required to establish representability.

To control the sizes of the coactivity complexes, we used only those periods of each neuron’s activity when it fired at least *m* spikes per coactivity window (i.e., time intervals defining simultaneity of neuronal activity, $w\approx 1/4$ secs; for justification of this value see^74,92^). Additionally, we used only those groups of coactive cells in which pairwise coactivity exceeded a threshold $\mu$ (Methods” section). Biologically, these selections correspond to using only the most robustly firing cells and cell assemblies for constructing the coactivity complexes^77^. The results demonstrate that majority of the coactivity complexes ${\mathcal {T}}$ computed for smallest possible *m* and $\mu$ exhibit low *persistent Leray dimensionality*, ${\bar{D}}_L =1$, which points at 2*D* representability of the underlying neuronal activity, with the Leray times $T_{L}$ similar to the corresponding learning times $T_{\min }$ (Fig. 2C). We also found that Eckhoff inequalities are typically satisfied throughout the navigation period, i.e., that the Eckhoff criterion does not significantly limit the scope of representable spiking in this case (Fig. 2D).

### Region connection calculus (RCC)

An independent perspective on spatial representability is provided by Qualitative Space Representation approach (QSR^93,94^), which sheds a new light on the dynamics of neuronal maps. From QSR’s perspective, a population of cells ${\mathfrak {C}}=\{c_1,c_2,\ldots ,c_N\}$ may represent a set of abstract, or *formal* spatial regions $R=\{r_1,r_2,\ldots ,r_N\}$, if the relationships between them, as defined by the cells’ coactivity, can be consistently actualized in a topological space *X* by a set of explicit regions, $\Upsilon =\{\upsilon _1, \upsilon _2,\ldots \upsilon _N\}$.

Specifically, regions $r_i$ and $r_j$ encoded by the cells $c_i$ and $c_j$ can be:

1. disconnected, $\mathsf {DR}(r_i,r_j)$, if $c_i$ and $c_j$ never cofire;
2. equal, $\mathsf {EQ}(r_i,r_j)$, if $c_i$ and $c_j$ are always active and inactive together;
3. proper part of one another, if $c_j$ is active whenever $c_i$ is, $\mathsf {PP}(r_i,r_j)$, or vice versa, $\mathsf {PPi}(r_i,r_j)$;
4. partially overlapping, $\mathsf {PO}(r_i,r_j)$, if $c_i$ and $c_j$ are sometimes (but not always) coactive.

**Figure 3:**
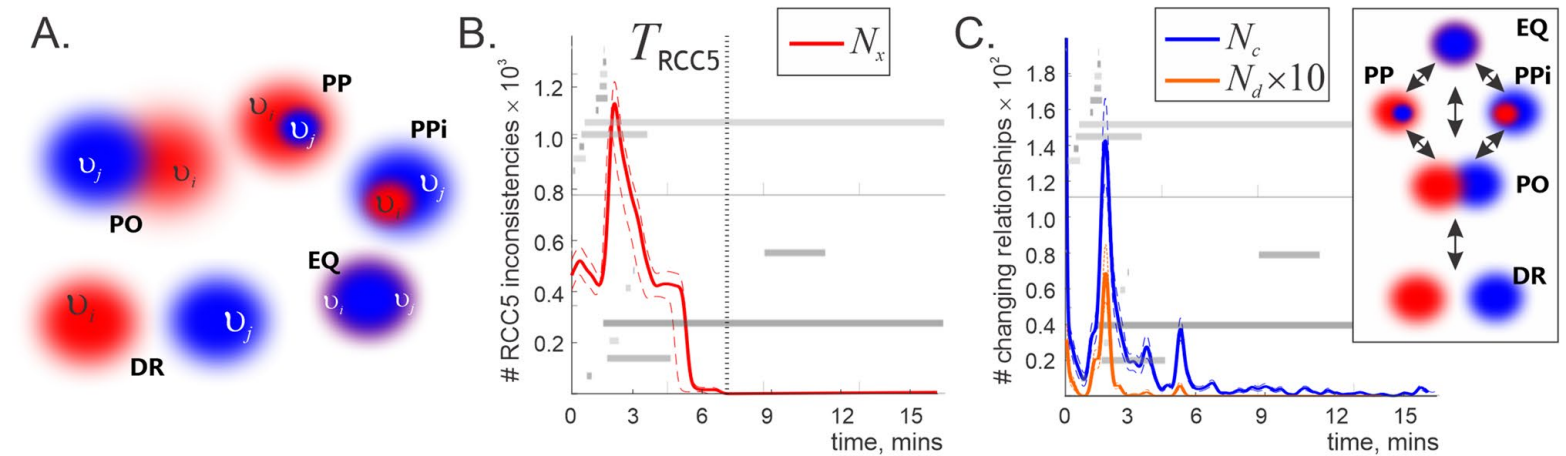
RCC5 analyses. (**A**) Two regions with soft boundaries, e.g. two firing fields $\upsilon _i$ and $\upsilon _j$, can overlap, $\textsf {PO}(\upsilon _i,\upsilon _j)$, be proper parts of each other, $\textsf {PP}(\upsilon _i, \upsilon _j)$ or $\textsf {PPi}(\upsilon _i,\upsilon _j)$, be disconnected $\textsf {DR}(\upsilon _i,\upsilon _j)$ or coincide $\textsf {EQ}(\upsilon _i,\upsilon _j)$. (**B**) Number $N_x(t)$ of inconsistent triples of RCC5 relationships appearing in the ${\mathcal {R}}_5(t)$ relational framework constructed for the same neuronal ensemble as illustrated in Fig. 2. The barcode diagram for the corresponding integrating coactivity complex (Fig. 2B) is shown in the background, to illustrate the correspondence between the RCC5 and the homological dynamics. $T_{{{\,\mathrm{{\textsf {RCC5}}}\,}}}$ (dotted line) marks the time when inconsistencies in the ${\mathcal {R}}_5(t)$ schema disappear. Results averaged over 10 repetitions, error margins shown by the dashed lines. (**C**) The net number of changes of RCC5-relationships between two subsequent moments of time, $N_c(t)$, shown by the blue line, and the number $N_d(t)$ of changes that violate the RCC5 continuity order (top right panel), shown by the orange line. For better illustration, $N_d(t)$ is scaled up by a factor of 10. Initially, discontinuous events are frequent but shortly before $T_{{{\,\mathrm{{\textsf {RCC5}}}\,}}}$ they disappear entirely, leaving the stage to qualitatively continuous sequences. The same barcodes are added in the background, error margins shown by dashed lines.

These five relations fully capture mereological configurations of regions in a first-order logical calculus known as RCC5 (Fig. 3A^95^). Using mereological (i.e., parthood-based see^96^ and Methods” section), rather than topological, distinctions reflects softness of the firing fields’ boundaries: the probabilistic nature of neuronal spiking does not warrant determining whether two regions actually abut each other or not.

A key property of a RCC5-framework defined by spiking neurons—a ${\mathcal {R}}_5$ schema—is its internal consistency^40^. It may turn out, e.g., that some pairs of cells encode relationships that are impossible to reconcile, e.g., $\mathsf {PP}(r_i,r_j)$, $\mathsf {DR}(r_j,r_k)$ and $\mathsf {PO}(r_i,r_k)$. Indeed, if an actual region $\upsilon _i$ is contained in $\upsilon _j$ then it cannot possibly overlap with a region $\upsilon _k$ that is disconnected from $\upsilon _j$. Correspondingly, the neuronal activity that produces such inconsistencies (for the full list see Table 1 in Methods” section) is not representable—not even interpretable in spatial terms. On the other hand, it can be shown that if all triples of relationships are consistent, then ${\mathcal {R}}_5(t)$ does possess a spatial model, i.e., there exists a set of regions $\upsilon _i$ (with no prespecified properties such as convexity, connectivity or dimensionality) that relate to each other as the $r_i$s relate in ${\mathcal {R}}_5$^93–99^.

To verify whether spiking activity is representable in this QSR sense, we constructed an inflating ${\mathcal {R}}_5(t)$-schema (an RCC5-framework growing as spiking data accumulates, similar to (2)) for each neuronal ensemble and counted the inconsistent triples of relationships at each moment *t*. The results show that all ${\mathcal {R}}_5(t)$-schemas start off with numerous inconsistencies, which tend to disappear after a certain period $T_{{{\,\mathrm{{\textsf {RCC5}}}\,}}}$ that is typically smaller than the Leray time $T_L$ (Fig. 3B,C).

**Table 1:** $\mathsf {RCC5}$ compositions. Given three regions, *x*, *y* and *z*, and two relationships ${\mathsf {R}}_1(x,y)$ and ${\mathsf {R}}_2(y,z)$, the relationship ${\mathsf {R}}_3(x,z)$ is not arbitrary. A map is consistent, if every triple of relationships is $\mathsf {RCC5}$–consistent.

$\circ$	$\mathsf {DR}(y,z)$	$\mathsf {PO}(y,z)$	$\mathsf {PP}(y,z)$	$\mathsf {PPi}(y,z)$	$\mathsf {EQ}(y,z)$
$\mathsf {DR}(x,y)$	$\mathsf {any}$	$\mathsf {DR, PO,PP}$	$\mathsf {DR, PO,PP}$	$\mathsf {DR}$	$\mathsf {DR}$
$\mathsf {PO}(x,y)$	$\mathsf {DR, PO,PPi}$	$\mathsf {any}$	$\mathsf {PO, PP}$	$\mathsf {DR, PO,PPi}$	$\mathsf {PO}$
$\mathsf {PP}(x,y)$	$\mathsf {DR}$	$\mathsf {DR, PO,PP}$	$\mathsf {PP}$	$\mathsf {any}$	$\mathsf {PP}$
$\mathsf {PPi}(x,y)$	$\mathsf {DR, PO,PPi}$	$\mathsf {PO, PPi}$	$\mathsf {PO, EQ,PP,PPi}$	$\mathsf {PPi}$	$\mathsf {PPi}$
$\mathsf {EQ}(x,y)$	$\mathsf {DR}$	$\mathsf {PO}$	$\mathsf {PP}$	$\mathsf {PPi}$	$\mathsf {EQ}$

The net dynamics of RCC5 relationships is illustrated on Fig. 3C. Note that some of these changes may be attributed to the regions’ continuous reshapings or displacements, e.g., two overlapping regions may become disconnected, $\textsf {PO}(r_i,r_j)\rightarrow \textsf {DR}(r_i,r_j)$, is $r_i$ moves away from $r_j$, or $r_i$ may move into $r_j$, inducing $\textsf {PO}(r_i,r_j)\rightarrow \textsf {PP}(r_i,r_j)$. In contrast, a jump from a disconnect to a containment, without an intermediate partial overlap, e.g., $\textsf {DR}(r_i,r_j)\rightarrow \textsf {PP} (r_i,r_j)$ rather than $\textsf {DR}(r_i,r_j) \rightarrow \textsf {PO}(r_i,r_j) \rightarrow \textsf {PP}(r_i,r_j)$, would be a discontinuous, abrupt change. As shown on Fig. 3C, discontinuous transitions are common at the initial stages of navigation, but shortly before $T_{{{\,\mathrm{{\textsf {RCC5}}}\,}}}$ they disappear, indicating that the relationships between regions encoded within a sufficiently well-developed ${\mathcal {R}}_5(t)$ schema evolve in a continuous manner.

These outcomes not only provide an alternative lower-bound estimate for the time required to accumulate data for producing low-dimensional spatial representations, but also help understanding the nature of processes taking place prior to Leray time. In particular, the exuberant initial dynamics, homologically manifested through an incipient outburst of spurious loops in the coactivity complexes (Fig. 2A,B, $t<T_{\min }$), cannot be interpreted as a mere “settling” of topological fluctuations in the cognitive map—according to Fig. 3B, the ${{\,\mathrm{{\textsf {RCC5}}}\,}}$-schema does not form a coherent topological stratum for $t<{\mathcal {T}}_{{{\,\mathrm{{\textsf {RCC5}}}\,}}}$. Rather, the initial disorderly period should be viewed as the time of transition from a nonspatial to a spatial phase, followed by spatial dynamics (for $t>{\mathcal {T}}_{{{\,\mathrm{{\textsf {RCC5}}}\,}}}$) that involves, *inter alia*, dimensionality reduction and other restructurings (Fig. 3C).

### Current summary

Taken together, these results show that even in the simplest “reactive” model, in which neuronal firings are simulated as responses to regular domains covering a compact space, the low-dimensional representability is not an inherent, but an emergent property. In particular, RCC5-analyses suggest that spatial interpretation of neuronal spiking becomes possible after a finite period. During the times that exceed both $T_{{{\,\mathrm{{\textsf {RCC5}}}\,}}}$ and $T_L$, the spiking data can be interpreted in terms of firing fields in a space *X* of dimensionality higher than the persistent Leray dimensionality of the corresponding coactivity complex, $\dim (X)>{\bar{D}}_L({\mathcal {T}})$.

Physiologically, this implies that the outputs of place cells, head direction cells, view cells, etc., may not be immediately interpretable by the downstream networks as representations of spatial regions—the information required for such inference appears only after a certain “evidence integration.” Correspondingly, the firing field maps constructed according to the standard experimental procedures^1,19,30^ also cannot be considered as automatic “proxies” of cognitive maps: such interpretations are appropriate only after the representability of the corresponding coactivity complexes is established. Another principal conclusion is that representability of the spiking activity depends not only on the spiking outputs, but also on how the information carried by these spikes is detected and processed. In particular, spikes integrated over extended periods are likelier to permit a consistent firing field interpretation than spikes counted via coactivity detection. On the technical side, these results imply that an accurate description of the firing fields’ plasticity should include possible dimensionality changes^101–103^.

### Multiply connected place fields

**Figure 4:**
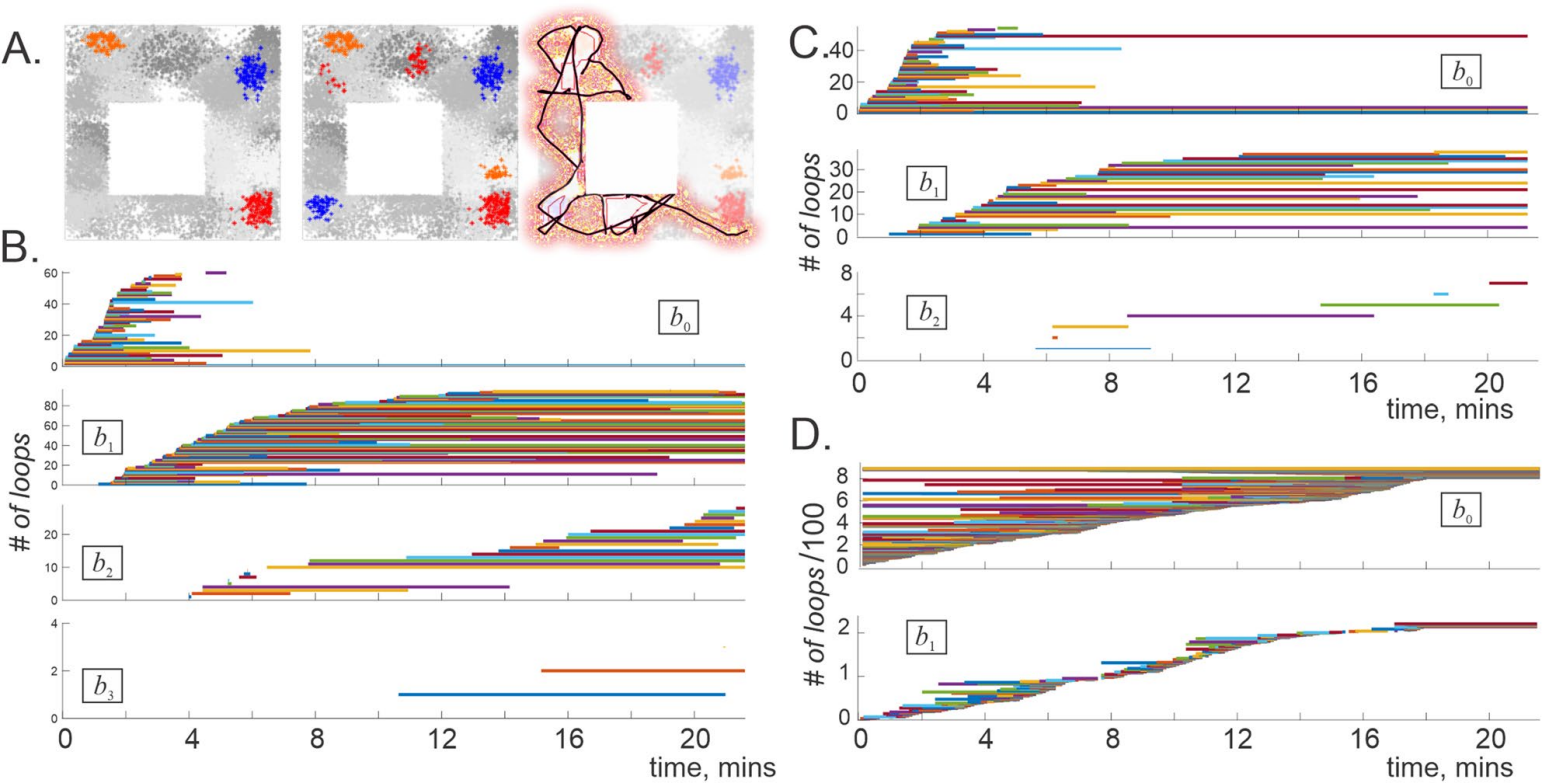
Topological dynamics in maps with multiple firing fields. (**A**) Left panel shows three examples of convex place fields used to obtain the results illustrated in Fig. 2. Allowing a cell to spike in several ($2-3$) locations produces multiply connected place fields (middle panel; clusters of dots of a given color correspond to spikes produced by a single simulated neuron). Right panel shows a $\varpi =50$ second long fragment of the trajectory $\gamma _{\varpi }$ covering a segment $\chi _{\varpi }$ of the environment (reddened area). (**B**) The Leray dimensionality of the detector-complex, evaluated for the same place cell population as in Fig. 2A, can reach $D({\mathcal {T}}_{\sigma })=4$ if we allow $30\%$ of multiply connected place fields ($2-3$ components each). (**C**) In a clique coactivity complex, the spurious loops in dimensions $D=2$ and lower may persist indefinitely, implying either that the firing fields are 3*D*-representable *or* that they may be multiply connected. Note that the number of spurious loops in both ${\mathcal {T}}_{\sigma }$ and in ${\mathcal {T}}_{\varsigma }$ is higher than in the case with convex firing fields (Fig. 2A,B). (**D**) The persistence bars computed for the flickering complex ${\mathcal {F}}_{\varpi }$ with spike integration window $\varpi =1$ minute, indicate stable mean Leray dimensionality $\langle D({\mathcal {F}}_{\varpi })\rangle =1$, implying that the local charts $\chi _{\varpi }$ are planar and hence that the firing fields are two-dimensional.

A key simplification used in the simulations described above is that firing fields were modeled as convex regions. While this assumption is valid in some cases^100^, multiply connected firing fields are also commonly observed (Fig. 4A^104,105^). From our current perspective, the issue is that multiple connectivity of the cover elements (3) may increase the Leray dimensionality of the corresponding nerve complex^49,106^ and thus bring additional ambiguity into the analyses. Identification of the firing fields’ connectivity from the spiking data is an elaborate task that requires tedious analyses of the spike trains produced by individual cells or cell groups over periods comparable to the Leray and the learning times^83,109^. To circumvent these difficulties, we reasoned as follows.

Suppose that the spiking activity used to produce a coactivity complex ${\mathcal {T}}(t)$ is generated as a moving agent (animal’s body, its head, its gaze) follows a trajectory $\gamma (t)$ over a space *X*, covered with stable firing fields $\upsilon _i$, $i=1,\ldots ,N$. Consider a navigation period $\varpi$ that spans over a smaller segment of this trajectory, $\gamma _{\varpi }=\{\gamma (t): t\in \varpi \}$. If $\varpi$ is sufficiently short, then one would expect $\gamma _{\varpi }$ to cross at most one component of a typical firing field $\upsilon _i$; even if $\gamma _{\varpi }$ meets more than one component of a multiply connected $\upsilon _i$, this property may not manifest itself in the resulting spike trains, i.e., $\upsilon _i$ should be *effectively* simply connected (Fig. 4A). Correspondingly, the Leray dimensionality of the coactivity complex acquired during that period should reflect the dimensionality of a small underlying fragment of *X*—a *local chart* $\chi _{\varpi }$—that contains $\gamma _{\varpi }$ (topologically, $\chi _{\varpi }(\gamma )\cong \{\cup _j\upsilon _j|\gamma _{\varpi } \cap \upsilon _j\ne \varnothing \}$). The dimensionality of $\chi _{\varpi }$ can then be ascribed to all the contributing $\upsilon _j$s, $\dim (\upsilon _j) =\dim (\chi _{\varpi })$.

Further, if the $\varpi$-period is allowed to shift in time, then the segment $\gamma _{\varpi }$ will also slide along the trajectory $\gamma (t)$; the spikes fired within each *t*-centered window, $\varpi _t = [t-\varpi /2,t+ \varpi /2]$, will then produce a $\varpi _t$-specific *flickering coactivity complex* ${\mathcal {F}}_{\varpi } (t)\subseteq {\mathcal {T}}(t)$, whose topological properties may change with time^78,110,111^. Since ${\mathcal {F}}_{\varpi }(t)$ contains a finite number of elements, it will reconfigure at discrete moments, $t_1,t_2,\ldots$, and remain unchanged in-between, ${\mathcal {F}}_{\varpi }(t)={\mathcal {F}}_{\varpi }(t_k)$, $t \in [t_k,t_{k+1})$. If a given instantaneous configuration ${\mathcal {F}}_{\varpi }(t_k)$ is representable, then its vertexes correspond to the regions comprising the local chart $\chi _{\varpi }(t_k)$, with dimensionality $\dim ( \chi _{\varpi }(t_k))\ge {\bar{D}}_L({\mathcal {F}}_{\varpi }(t_k))$. If two such complexes overlap, ${\mathcal {F}}_{\varpi } (t_k)\cap {\mathcal {F}}_{\varpi }(t_{l})\ne \varnothing$ (i.e., their vertex sets overlap), then their respective charts also overlap $\chi _{\varpi }(t_k)\cap \chi _{\varpi }(t_{k+1})\ne \varnothing$, which allows relating their topological properties, including properties of the representing regions.

Clearly, the outcome may depend on how each $\gamma _{\varpi }$ is embedded into *X*, the spiking parameters, etc. Moreover, since the Leray dimensionality of the instantaneous complexes can change, so can the dimensionalities of the corresponding local charts: ${\bar{D}}_L({\mathcal {F}}_{\varpi }(t_k))\ne {\bar{D}}_L({\mathcal {F}}_{\varpi }(t_l))$ may entail $\dim (\chi _{\varpi }(t_k))\ne \dim (\chi _{\varpi }(t_l))$. This may seem as a contradiction since the representing space is naturally assumed to be a topological manifold, i.e., all of its local charts, arbitrarily selected, should have the same dimensionality $\dim (\chi _{\varpi }(t))=\dim (X)=D$. On the other hand, the deviations of the local dimensionality estimates from a fixed *D* can be viewed as mere fluctuations caused by occasional contribution of multiply connected firing fields or by other noise sources, e.g., by stochasticity of neuronal spiking^112^. One can hence attempt to discover the true dimensionality of *X* by evaluating the mean Leray dimensionality of the instantaneous complexes,

$$\begin{aligned} \dim (X)=\langle {\bar{D}}_L({\mathcal {F}}_{\varpi }(t_k))\rangle _k, \end{aligned}$$

which physiologically alludes to learning the physical structure of the underlying space from the recurrent information.

Numerical verification of the viability of the proposed approaches can be achieved by simulating multiply connected firing fields and computing homological dynamics of the resulting coactivity complexes. To that end, we randomly added $2-3$ additional convex components to $\sim 30\%$ of the place fields (Fig. 4A) and simulated the topological dynamics of the corresponding complexes.

The results show that multiple connectivity of the firing fields does indeed increase Leray dimensionality in both the detector and the integrator complexes, ${\mathcal {T}}_{\sigma }(t)$ and ${\mathcal {T}}_{\varsigma }(t)$. Moreover, in contrast with the complexes generated off the maps with convex fields, the maps with multiply connected fields tend to produce persistent higher-dimensional loops, notably in the coactivity detecting complexes ${\mathcal {T}}_{\sigma }(t)$ (compare Figs. 2A and  4B). In the spike integrating clique complex ${\mathcal {T}}_{\varsigma }(t)$, the Leray dimensionality remains low and may in some cases retain the physical value $D_{L}({\mathcal {T}}_{\varsigma })=1$, although topological loops in dimensions $D=2$ and even higher may also appear (Fig. 4C). Thus, multiple firing field connectivity significantly increases the number of spurious 1*D* holes (by 200–300$\%$), precluding both types of complexes from assuming the physically expected topological shapes.

Tighter dimensionality estimates can be produced by using shorter spike integration windows $\varpi \lesssim T_{\min }$ and constructing flickering coactivity complexes ${\mathcal {F}}_{\varpi }(t)$ from pairwise coactivities detected over $\varpi$-periods shifting by discrete steps $\Delta \varpi$ and yielding an array of windows $\varpi _1,\varpi _2,\varpi _3,\ldots$ centered at $t_k =\varpi /2+(k-1)\Delta \varpi$. The specific $\varpi$-values were chosen comparable to the characteristic time required by the rat to run through a small segment of the environment: $\varpi \approx$ 25–65 s for the arena shown on Figs. 1A and 4A. The Betti numbers for this case were evaluated using zigzag homology theory—a generalization of the persistent homology theory that applies to complexes that can not only grow, but also shrink, break apart, fuse back again, etc.^113,114^. In particular, this approach allows studying how the topological fluctuations in ${\mathcal {F}}_{\varpi }(t)$ affect its Leray dimensionality ${\bar{D}}_L({\mathcal {F}}_{\varpi }(t))$ from moment to moment.

Typical results illustrated on Fig. 4D show that there appears a large number of spurious 0*D* loops—disconnected pieces—with lifetimes nearly exponentially distributed about the learning periods $T_{\min }^{\varsigma }$, which suggests that fragments of ${\mathcal {F}}_{\varpi }(t)$ appear and disappear at random over such periods. The transient 1*D* loops also form and decay at $\varpi$-timescale. However, the most important outcome is that the topological dynamics in dimensions $D>1$ trivializes—the higher dimensional loops in ${\mathcal {F}}_{\varpi }$ occur very rarely, if ever. These properties are qualitatively unaffected by varying the discretization step $\Delta \varpi$ ($\varpi /20\lesssim \Delta \varpi \lesssim \varpi /10$) or changing the window width $\varpi$, i.e., the estimates of the mean Leray dimensionality $\langle {\bar{D}}_L({\mathcal {F}}_{\varpi })\rangle =1$ are stable and reveal physical planarity of the representing space.

Verification of the RCC5-consistency of the spiking data produces the same qualitative results as in the case with simply connected firing domains: the ${\mathcal {R}}_5(t)$-schemas become consistent after a learning period $T_{{{\,\mathrm{{\textsf {RCC5}}}\,}}}< T_{\min }^{\varsigma }$, upon which neuronal activity becomes spatially interpretable, and, by the Leray and Eckhoff arguments, representable in dimensions $D\ge \langle {\bar{D}}_L({\mathcal {F}}_{\varpi }) \rangle$.

### Electrophysiological data

**Figure 5:**
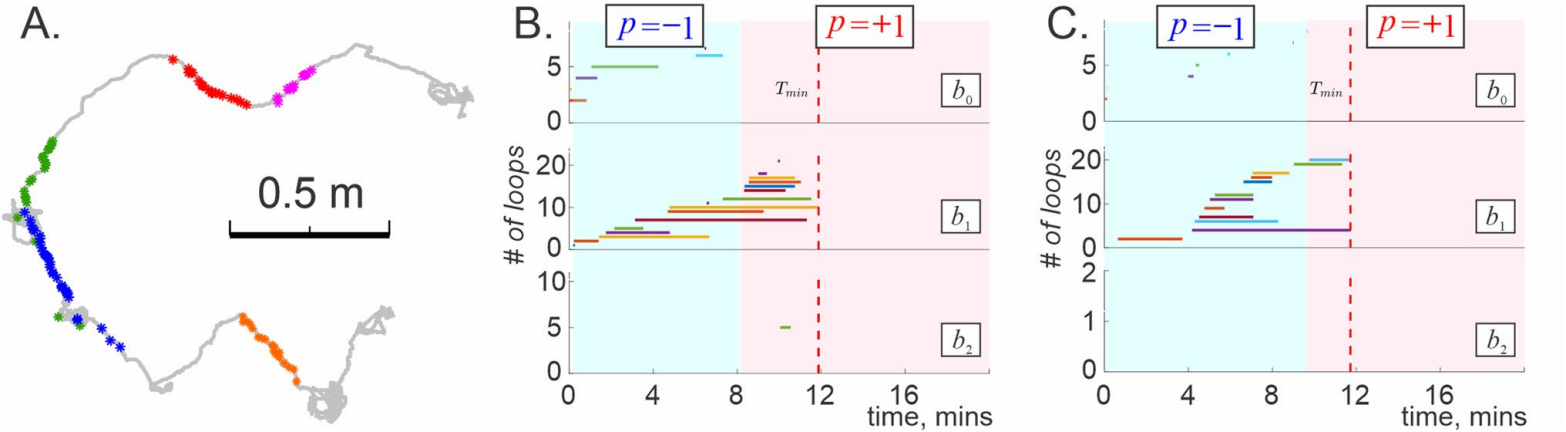
Multiple firing fields. (**A**) Spikes produced by five place cells (dots of different color) recorded in hippocampal CA1 area of a rat navigating a solid U-shaped groove with hard walls (speed $v\ge 3$ cm/sec). Since the rat could move from place to place in strict sequence, this environment is topologically one-dimensional. The underlying gray line shows a fragment of the rat’s trajectory (for more details see^115^). (**B**) Spurious topological loops in the corresponding coactivity complex disappear in $T_L\approx 12$ minutes, revealing persistent Leray dimensionalities ${\bar{D}}=0$. The blue background highlights the period during which the coactivity complex computed using only cells with convex place fields is not 1*D* representable ($p=-1$). The transition to $p(t)=+1$, marking the onset of 1*D* representability occurs at a time close to $T_L$. (**C**) Topological dynamics of the coactivity complex constructed using the data recorded during the outbound moves only shows qualitatively similar behavior.

We applied the analyses described above to spiking activity recorded in the hippocampus (CA1 area) of rats navigating a linear environment shown on Fig. 5A (for more data description and experimental specifications see^115^). A typical running session, during which the animal performed $45-70$ laps between the tips of the track, provided $N_c\lesssim 25$ simultaneously recorded neurons, allowing to construct small coactivity complexes that quickly become RCC5-consistent, comply with the Eckhoff conditions, and exhibit persistent Leray dimensionality, ${\bar{D}}_L=0$, with typical persistent Leray time $T_L\approx 10$ mins (Fig.5B).

The vanishing ${\bar{D}}_L$ indicates that a linear track illustrated in Fig. 5A is contractible and implies 1*D*-representability. The latter can also be tested independently via RCC5 analyses, which in this case allows identifying the track’s linear structure^116^.

Since some of the hippocampal place fields are multiply connected, we also applied sliding window analyses, adjusting the spike integration period $\varpi _i$ to match the duration of the animal’s $i^{\text {th}}$ run from one end of the track to the other (typically $2\le \varpi _i \le 10$ secs). Computations reveal that the resulting complexes ${\mathcal {F}}_{\varpi _i}(t)$ exhibit the same mean Leray dimensionality $\langle {\bar{D}}_L({\mathcal {F}})\rangle =0$, which is consistent with the persistent ${\bar{D}}_L$ estimates above. Combining these results produces convergent evidence that in this case the hippocampus does indeed map out a 1*D* spatial domain (rather than 2*D*, see^115^).

The latter conclusion can, in fact, be verified by yet another representability test, which applies only to 1*D* cases and presumes firing field convexity. The Golumbic-Fishburn (GF) algorithm^53–57^ is based on computing a binary index *p*: the 1*D*-representable simplicial complexes $\Sigma$ yield $p(\Sigma )=+1$, and the non-representable complexes produce $p(\Sigma )=-1$. For the inflating or flickering coactivity complexes this index becomes time-dependent, $p=p(t)$, marking the evolution of 1*D* representability (Methods” section). Applying the GF-algorithm to the inflating coactivity complexes constructed for cells with convex place fields only, we found that 1*D* representability, $p(t)=+1$, appears in about $T_{+}\approx 10$ mins, close to the Leray time (Fig. 5B,C), demonstrating consistency with the previously obtained results.

Lastly, we addressed a particular property of the place cell’s spiking activity in linear environments—the place fields’ directionality: a given place cell may fire during the outbound, but not inbound directions, or vice versa^117^. We verified that the topological dynamics exhibited by the coactivity complexes built using only the outbound or only the inbound activity are very similar to the dynamics of the full (bidirectional) complex ${\mathcal {T}}_{\varsigma }(t)$ (Fig. 5B,C), implying that place cell directionality does not necessarily compromise 1*D* representability of spiking activity.

## Discussion

Topological analyses of the spiking data allow testing whether a given type neuronal activity may arise from a “spatial map,” i.e., whether each neuron’s spiking marks a domain similar to a place field, a head direction field, a view field, etc., in a certain low-dimensional space. Thus far, establishing correspondences between neurons and firing fields was based on matching the spike trains with spatial domains empirically, through trial and error^1,19,30^. Here we attempt to address this question in a principled way, through intrinsic analyses of the spiking data, without presuming or referencing *ad hoc* constructions. A set of hands-off algorithms discussed above allows objective estimates for the dimensionality of a space needed to model the patterns of neuronal firing—a method that is unaffected by technical limitations, experimental ingenuity or complexity (e.g., nonlinearity) of the required firing field arrangements.

To follow the dynamics of the coactivity complexes we extend the conventional approaches of representability theory into the temporal domain, obtaining several complementary time-dependent markers of representability. In particular, we use persistent homologies to extend Leray’s theory to the case of inflating simplicial complexes and zigzag homologies in the case of flickering simplicial complexes. The latter approach is especially valuable as it allows extracting stable topological information from spiking data that may be generated from the maps with multiply connected firing fields or encumbered by other inherent irregularities, in spirit with the general ideas of topological persistence^68–73^. It should also be mentioned that mathematical discussions of the persistent nerve theorem, alternative to ours and more formal, have began to appear^118,119^; however at this point our studies are independent.

A principal observation suggested by our analyses is that representability is a dynamic, emergent property that characterizes current information supplied by the neuronal activity. Moreover, representability depends not only on the amount and the quality of the spiking data itself, but also on the mechanisms used for processing and interpreting this data. Both aspects affect the time required to establish the existence of a representing space and its dimensionality. An implication of this observation is that experimentally constructed firing field maps (place field maps, head direction maps, etc.) cannot be automatically regarded as direct models of cognitive representations of ambient spaces^15–21^ or more general spatial frameworks^120^; correctness of such interpretations may require more nuanced considerations.

## Methods

### Physiological parameters and constructions

- *Simulated trajectory* $r(t)=(x(t),y(t))$, used for generating coactivity complexes was obtained by modeling a rat’s non-preferential exploratory behavior—navigation without favoring of one segment of the environment ${\mathcal {E}}$ over another (Fig. 1A). The mean speed of about $\sim 20$ cm/sec was selected to match experimentally recorded speeds. The direction of the velocity $v(t)=(v_x(t),v_y(t))$ defines the “angular trajectory” $\varphi (t)=\arctan {v_y(t)/v_x(t)}$ that traverses the space of directions, $S^1$, allowing to simulate head direction cell activity as the rat explores ${\mathcal {E}}$^48,74,82^. The simulated *navigation period*, $T = 25$ minutes, was selected to match the duration of a typical “running session” in electrophysiological experiments^100^. A shorter *spike integration window* $\varpi \ll T$ was used to limit the pool of spiking data for time-localized computations.
- *Poisson spiking rate* of a place cell *p* depends on the animal’s location *r*(*t*),
  $$\begin{aligned} \lambda _p(r)=f_{p}e^{-\frac{|r-r_p|^2}{2s^2_{p}}}, \end{aligned}$$
  where $f_p$ is the cell’s maximal firing rate and $s_p$ defines the size of its place field^101^. A similar formula defines the firing rate of a head direction cell *h*, $\lambda _h(\varphi )$, as a function of the animal’s ongoing orientation $\varphi$, the cell’s preferred orientation angle $\varphi _h$, its maximal rate $f_h$ and the size of its preferred angular domain $s_h$. In all simulations the firing fields were stable, i.e., the parameters of $\lambda _c$ and $\lambda _h$ remained constant.
- *Neuronal ensembles* produce lognormal distributions of the maximal firing rate amplitudes, $f_c$, and of the firing field sizes, $s_c$^48,121^. We tested about 17, 000 different ensembles, in which the ensemble mean maximal rate *f* ranged between 4 and 40 Hz for the place cells and between 5 to 35 Hz for the head direction cells. The ensemble mean firing field sizes varied between 10 to 90 cm for the place fields and between $12^{\circ }$ and $36^{\circ }$ degrees for the angular fields. For all ensembles, the firing field centers were randomly scattered over their respective representing spaces.
- *Multiple Firing Fields* were generated by adding two or three randomly scattered auxiliary spiking centers $r_{c'}$, $r_{c''}$, etc.,
  $$\begin{aligned} \lambda _c(r)=f_{c}e^{-\frac{|r-r_{c}|^2}{s^2_{c}}}+f_{c'}e^{-\frac{|r-r_{c'}|^2}{s^2_{c'}}}+\ldots \,\,\, . \end{aligned}$$
  The maximal firing rates at the auxiliary locations are smaller than the rate at the main location, $f_c>f_{c'}>\ldots$, as suggested by the experiments^104,105^.
- *The activity vector* of a cell, $m_{c}=[m_{c,1},\ldots , m_{c,n}],$ is constructed by binning its spike trains into $w=1/4$ seconds long “coactivity windows”^74,92^. Each $m_{c,k}$ specifies how many spikes were fired by *c* into the $k^{th}$ time bin, *n* is defined by the duration of navigation, $n=\lfloor T/w\rfloor$. High activity periods can be identified by selecting time bins in which the number of fired spikes exceeds an activity threshold *m*.
- *Coactivity*. Two cells, $c_i$ and $c_j$, are *coactive* over a time period *T*, if the formal dot product of their activity vectors does not vanish, $m_{ij}(T)=m_{c_i}(T) \cdot m_{c_j} (T)\ne 0$. The set of all pairwise coactivities forms the coactivity matrix $M(T)=\Vert m_{ij}(T)\Vert$. Highly coactive pairs of cells are the ones whose coactivity exceeds a threshold $\mu$.

### Topological propaedeutics

#### Graphs

**Figure 6:**
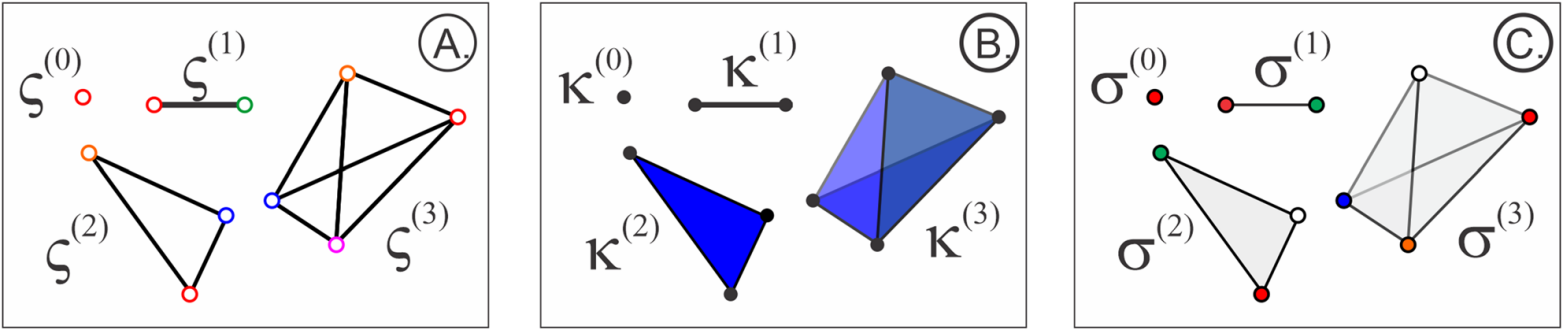
Cliques and simplexes. (**A**) Pairwise interlinked subsets of vertexes in graph *G* form its cliques. Shown is a vertex $\varsigma ^{(0)}$ (0-clique), a link $\varsigma ^{(1)}$ (1-clique), a three-vertex $\varsigma ^{(2)}$ and a four-vertex $\varsigma ^{(3)}$ cliques. (**B**) Geometric simplexes are actual geometric figures: a 0*D* dot ($\kappa ^{(0)}$), a 1*D* link ($\kappa ^{(1)}$), a 2*D* triangle ($\kappa ^{(2)}$) and a 3*D* tetrahedron ($\kappa ^{(3)}$). (**C**) The corresponding abstract simplexes are simply ordered sets of vertexes: $\sigma ^{(0)}$ (single vertex), $\sigma ^{(1)}$ (pairs of vertexes), $\sigma ^{(2)}$ (triples) and $\sigma ^{(3)}$ (quadruples).

- *A graph* *G* is defined by its vertices, $V=\{v_1,v_2,\ldots ,v_n\}$, and a set of edges *E* that link certain pairs of vertexes. A formal description of a graph is given by its connectivity matrix *C*(*G*), with the elements
  $$\begin{aligned} C_{ij}(G)= {\left\{ \begin{array}{ll} 1, \,\,\, &{} \hbox{if }v_i\hbox { and }v_j\hbox { are connected by edge }e_{ij}, \\ 0 \,\,\, &{} \hbox {if }v_i\hbox { and }v_j\hbox { are disconnected}. \end{array}\right. } \end{aligned}$$
- *A coactivity Graph* ${\mathcal {G}}$ is built by establishing functional links between cells that exhibit high activity and coactivity ($m_{c,k}\ge m$, $m_{ij}\ge \mu$ see above)^89,90^.
- A *clique* of order *d*, $\varsigma ^{(d)}$ in a graph is a fully interconnected subset of $(d+1)$ vertexes $v_{i_0},v_{i_1},\ldots ,v_{i_d}$ (Fig. 6A).
- Given a graph *G*, its *complement graph* ${\tilde{G}}$ is produced by flipping 0s and 1s in the connectivity matrix *C*(*G*), i.e., joining the disconnected vertexes of *G* and removing the existing edges.

#### Simplicial complexes

- *Geometric simplexes* are points (0-simplexes, $\kappa ^{(0)}$), line segments (1-simplexes, $\kappa ^{(1)}$), triangles (2-simplexes, $\kappa ^{(2)}$), tetrahedra (3*D*-simplexes, $\kappa ^{(3)}$), as well as their $d>3$-dimensional generalizations (Fig. 6A). Note that the set of vertexes opposite to a given vertex in a *d*-simplex $\kappa ^{(d)}$ spans a $(d-1)$-simplex—a *face* of $\kappa ^{(d)}$. The boundary of a *d*-simplex then consists of $(d+1)$ faces $\kappa ^{(d-1)}_1,\kappa ^{(d-1)}_2,\ldots , \kappa ^{(d-1)}_{d+1}$ (Fig. 6B).
- *Geometric simplicial complexes* are combinations of geometric simplexes that match each other vertex-to-vertex, so that a non-empty intersection of any two simplexes in *K* yields another *K*-simplex: if $\kappa _1,\kappa _2\in K$, then $\kappa _1\cap \kappa _2=\kappa _3\in K$.
- The collection of all simplexes of dimensionality *d* and less forms the *d*-*skeleton* of *K*, $sk_d(K)$.
- Topological analyses of simplicial complexes do not address simplexes’ shapes and are based entirely on the combinatorics of the vertexes shared by the simplexes. This motivates using *abstract simplexes* and *abstract simplicial complexes* that capture the combinatorial structure of $\kappa ^{(d)}$s without making references to their geometry. Specifically, an abstract 0-simplex is a vertex $\sigma ^{(0)}_i\equiv v_i$, an abstract 1-simplex is a pair of vertexes, $\sigma ^{(1)}_{ij}=[v_i,v_j]$; an abstract 2-simplex is a triple of vertexes, $\sigma ^{(2)}_{ijk}=[v_i,v_j,v_k]$, and so forth (Fig. 6C). Thus, abstract complexes may be viewed as multidimensional generalizations of graphs or as abstractions derived from the geometric simplicial complexes.

A *d*-element subset of an abstract *d*-simplex $\sigma ^{(d)}$ forms its $(d-1)$-face. The“face-matching” of the abstract simplexes in $\Sigma$ means simply that a nonempty overlap of two simplexes $\sigma _1,\sigma _2\in \Sigma$ is a simplex of the same complex, $\sigma _1\cap \sigma _2=\sigma _3\in \Sigma$. The latter property is commonly used to define abstract simplicial complexes for arbitrary sets, using families of their subsets that are closed under the “$\cap$” operation^41^.

**Figure 7:**
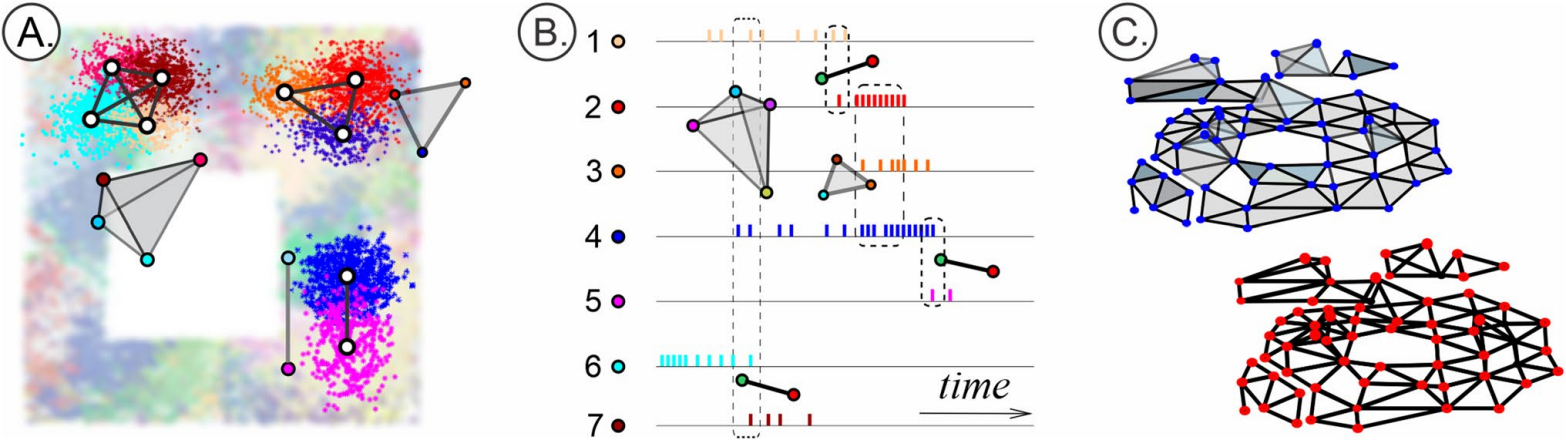
Cliques and simplexes. (**A**) Pairwise interlinked place fields produce cliques of the coactivity graph ${\mathcal {G}}$. Shown is a vertex $\varsigma ^{(0)}$ (0-clique), a link $\varsigma ^{(1)}$ (1-clique), a three-vertex $\varsigma ^{(2)}$ and a four-vertex $\varsigma ^{(3)}$ clique. (**B**) Geometric simplexes: a 0*D* dot ($\kappa ^{(0)}$), a 1*D* link ($\kappa ^{(1)}$), a 2*D* triangle ($\kappa ^{(2)}$) and a 3*D* tetrahedron ($\kappa ^{(3)}$). (**C**) The corresponding complexes: a simplicial coactivity complex ${\mathcal {T}}_{\sigma }$ whose simplexes (1) are detected as singular coactivity events (left) may topologically differ from the clique coactivity complexes ${\mathcal {T}}_{\varsigma }$, assembled from the cliques of a coactivity graph ${\mathcal {G}}$ (right) over a spike integration period $\varpi$. A simplicial complex *K* is a combination of matching simplexes. The set of vertexes and black lines highlight the 1*D*-skeleton of $sk_1(K)$.

- *Example 1*: The set of overlapping regions (4) define abstract simplexes (5) of the nerve complex (6) (Fig. 7A).
- *Example 2*: The combinations of coactive cells define coactivity simplexes (1), which together form a coactivity complex (Fig. 7B).
- *Example 3*. Vertexes of geometric simplexes that form a geometric simplicial complex *K* define abstract simplexes that form the corresponding abstract simplicial complex $\Sigma$ (Fig. 7C).
- The set of *d*-dimensional simplexes of a complex $\Sigma$ forms its (abstract) *d*-skeleton, $sk_d(\Sigma )$.
- *A clique complex* of an undirected graph *G* is an abstract simplicial complex formed by the cliques (fully interconnected subgraphs) of *G*^91^, Fig. 6A. Combinatorial properties of cliques are the same as simplexes’: a subset of a clique’s vertexes form a clique, overlap of two *G*-cliques is also a clique, $\varsigma _1\cap \varsigma _2=\varsigma _3\in G$ (Fig. 6). Thus, any graph *G* defines a unique clique complex ${\tilde{\Sigma }}(G)$. Note, that the 1-skeleton of a clique complex yields its underlying graph, ${{\,\mathrm{sk}\,}}_1({\tilde{\Sigma }}(G))=G$, but if $\Sigma$ is not a clique complex, then the clique complex built over its 1-skeleton does not reproduce $\Sigma$.
- *Coactivity complexes* used in this study are of two kinds. The first kind is formed by the abstract complexes ${\mathcal {T}}_{\sigma }$ built from simultaneously coactive cell groups (1). The second kind is formed as the clique complexes of the coactivity graphs ${\mathcal {G}}$^77,80^. The graph (co)activity thresholds *m* and $\mu$ are used to control the size of the complex ${\mathcal {T}}_{m,\mu }={\mathcal {T}}({\mathcal {G}}_{m,\mu })$: selecting $m\ge 2$, $\mu =1$ for small maps (i.e., counting cells that produce at least two spikes per time bin *w*) and $m\ge 2$, $\mu \ge 5$ for larger maps allows computing the full simplicial complex with dimensionality $\dim ({\mathcal {T}}_{m,\mu })\le 10$, for which we can numerically apply the Javaplex software^122^.

### Mereology

Mereology is the theory of parthood—relations between whole and part, as well as relations between parts within a whole^96,123^. Mereological level of describing spatial regions is cruder than topological, which also includes contact relationships. Since the latter is generally not captured by spiking data^40,116^, we use mereological RCC5 theory^95^.

#### Topological invariants

- *Homological groups* are designed to “count pieces” in a space *X* with suitable coefficients. The key property of these groups is that they remain unchanged—*invariant*—as *X* is continuously deformed (see^41,42^ for a gentle introduction to the subject). If the coefficients form an algebraic field *F*, then the homological groups, commonly referred to as the “homologies” of *X* are simply vector spaces $H_{0}(X,F),H_{1}(X,F),\ldots$, associated with *X* (one per dimension). Homologies can be easily computed for spaces whose “pieces” are explicitly defined, e.g., for the simplicial complexes, thus providing a way of identifying their topological structures. In practice, it is easier to use just the dimensionalities of $H_{*}$s—the *Betti numbers* $b_k= \dim (H_k(X,F))$, to count numbers of connectivity components, cavities, tunnels and other topological features of *X* in different dimensions^41,42^. For example, if *X* is the boundary of a hollow triangle (or another noncontractible 1*D* loop), then $\beta _1(X)=1$. If *X* is 1-dimensional complex, i.e., a graph, then $\beta _1(X)$ equals to the number of cycles in *X*, counted up to topological equivalence. If the triangle is “filled”, then it can be continuously contracted into a 0*D* point; since the latter has no topological structure in dimensions $d>0$, the corresponding Betti numbers also vanish. By the same argument a “filled” tetrahedron has $\beta _{k>0}=0$, but if the tetrahedron is hollow, then its boundary, being a 2*D* noncontractible loop (topologically—a 2*D* sphere) produces $\beta _2=1$, $\beta _{k>2}=0$. Similarly, for any *d*-simplex $\beta _{k>0}(\sigma ^{(d)}) = 0$, whereas for its hollow boundary, $\partial \sigma ^{(d)}$, the Betti numbers are $\beta _{d-1}(\partial \sigma ^{(d)})=1$, $\beta _{k\ne 0,d-1}(\partial \sigma ^{(d)})=0$ (Fig. 6). Same results apply to the “abstract” counterparts of all these complexes. Note also that continuous deformations of a 0*D* point *x* (a 0*D* topological loop) amount to “sliding” *x* inside of a space *X* that contains *x*; thus $\beta _0(X)$ simply counts such “sliding domains”, i.e., the number of connected components in *X*. As a result, all simplexes and simplicial complexes that consist of one piece have $\beta _0(X)=1$.
- *Persistent homology* theory allows tracing the topological structure in a filtered family of simplicial complexes, e.g., describing the topological dynamics of the inflating family (2)^70–73^. The Betti numbers plotted as function of the filtration parameter (in our case it is time, *t*) form the *barcode*, ${\mathfrak {b}}({\mathcal {T}},t)= (b_0({\mathcal {T}},t),b_1({\mathcal {T}},t),\ldots )$, which provides the exact mathematical meaning to the term “topological shape” used throughout the text. Each bar in ${\mathfrak {b}}({\mathcal {T}},t)$ can be viewed as the corresponding topological loop’s timeline^48,74–77,80^.
- *Zigzag Homology* theory allows tracking the Betti numbers of the “flickering” complexes—the ones whose simplexes can not only appear, but also disappear (see^113,114^ and Supplement in^110^). In particular, Zigzag homology techniques allow capturing the times when individual loops appear in the flickering complex, how long they persist, when they disappear, reappear again, etc.

#### Representability

A generic algorithm for checking whether a given complex can be built as a nerve of a *D*-dimensional cover is known only for $D=1$ (see below). However, there exist criteria that allow ruling out certain non–representable cases.

**Figure 8:**
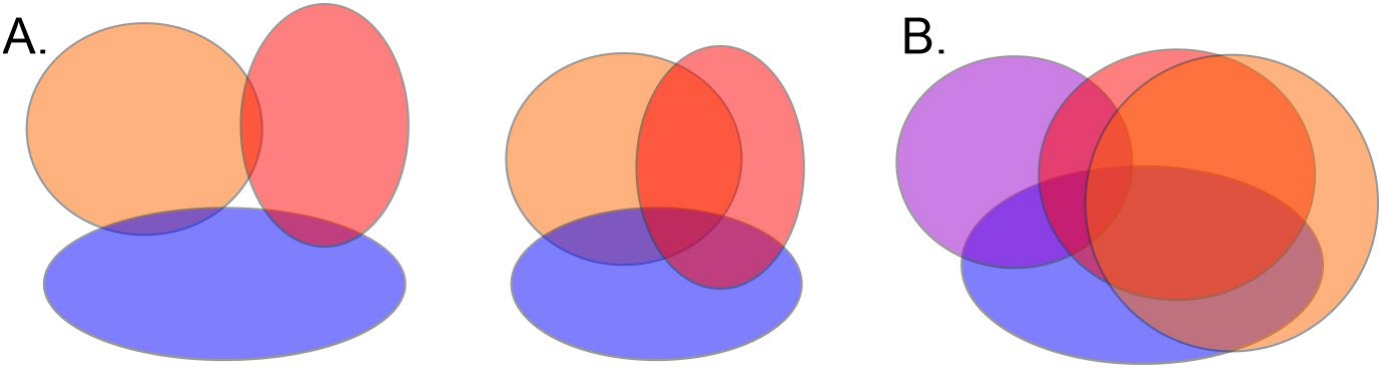
Helly’s theorem. (**A**) Three regions may exhibit both pairwise (left) or triple overlap (right). (**B**) The Helly number of a family of convex regions in ${\mathbb {R}}^d$ does not exceed $d+1$. Thus, for convex planar regions having all triple overlaps implies having all the higher order (i.e., for this particular picture quadruple) overlaps. Hence, the intersection patterns of convex planar subspaces are completely determined by the intersection patterns of triples.

- The *Leray criterion* posits that if a complex $\Sigma$ is a nerve of a *D*-dimensional cover with contractible overlaps (4), then its rational homologies in dimensions higher or equal than *D* should vanish, $H_{i\ge D}(\Sigma ,{\mathbb {Q}})=0$^58^. Moreover, homologies of all the subcomplexes $\Sigma ' \subseteq \Sigma$, induced by selecting vertex subsets of $\Sigma$ should also vanish, $H_{i\ge D}(\Sigma ', {\mathbb {Q}})=0$. These properties can be verified by computing the Betti numbers and verifying that $b_{i\ge D}(\Sigma ,{\mathbb {Q}})=0$. In practice, it is more convenient to carry out the computations over a finite field, such as ${\mathbb {Z}}_2$. Although the $b_k(\Sigma ,{\mathbb {Q}})$ numbers may in general differ from the $b_k(\Sigma ,{\mathbb {Z}}_2)$ numbers, the latter also have to obey the Leray condition and produce the same Leray dimensionality. As an example, the Leray condition poses that the boundary of the triangle is not 1-representable ($\beta _1(\partial \sigma ^{(2)}, {\mathbb {Z}}_2)>0$), but the triangle itself may be ($\beta _1(\sigma ^{(2)},{\mathbb {Z}}_2)=\beta _2(\sigma ^{(2)},{\mathbb {Z}}_2)=0$); the boundary of a tetrahedron is not 2-representable ($\beta _2(\partial \sigma ^{(3)},{\mathbb {Z}}_2)>0$), but the tetrahedron may be.
- *Amenta’s theorem* connects the Leray dimensionality of a simplicial complex to its *Helly number*, defined as follows. Let $\Upsilon =\{\upsilon _1,\upsilon _2,\ldots ,\upsilon _n\}$ be a finite family of regions (Fig. 8). The Helly number $h=h(\Upsilon )$ of the family is defined to be the maximal number of non-overlapping regions, such that every $h-1$ among them overlap. For the corresponding nerve complex ${\mathcal {N}}(\Upsilon )$, $h=h({\mathcal {N}})=h(\Upsilon )$ is the number of vertices of the largest simplicial hole in ${\mathcal {N}}$ (i.e., the dimension of the hole plus 2^49^). This observation can be used to attribute a Helly number to any simplicial complex $\Sigma$, $h(\Sigma )$. From the perspective of representability analyses, a key property of the Helly numbers is that they do not exceed $d+1$ for a *d*-Leray complex^49^. In particular, if the regions $\upsilon _i\in \Upsilon$ consist of up to *k* compact, convex domains in $R^d$, and any intersection $\upsilon _{i_1} \cap \dots \cap \upsilon _{i_t}$ also satisfies this property, then $h(\Upsilon )\le k(d+1)$^49,106,107^.
- *Eckhoff’s conjecture*. The *f*-vector $f=(f_1,f_2,\ldots ,f_n)$ of a simplicial complex $\Sigma$ is the list of numbers of its *k*-dimensional simplexes, $f_k=\#\{\sigma _i\in \Sigma |\dim (\sigma )=k\}$ (“*f*” is a traditional notation that should not be confused with the firing rates). The *h*-vector of $\Sigma$ is defined as
  $$\begin{aligned} h_k= {\left\{ \begin{array}{ll} f_k, \,\,\, &{} \text{ for } \,\,\,k=0,1,\ldots ,D-1, \\ \sum _{j\ge 0} (-1)^j {{k+j-D}\atopwithdelims (){j}} f_{k+1}, \,\,\, &{} \text{ for } \,\,\, k=D,D+1,\ldots \, , \end{array}\right. } \end{aligned}$$
  where parentheses denote the binomial coefficients. Given the combinatorial decomposition of *l*,
  8 $$\begin{aligned} l={{l_k}\atopwithdelims (){k}}+{{l_{k-1}}\atopwithdelims (){k-1}}+\ldots +{{l_j}\atopwithdelims (){j}}, \end{aligned}$$
  where $l_k\ge l_{k-1}\ge \ldots \ge l_j\ge j\ge 1$^108^, define the set of numbers
  $$\begin{aligned} l^{(k)}={{l_k}\atopwithdelims (){k-1}}+{{l_{k-1}}\atopwithdelims (){k-2}}+\ldots +{{l_j}\atopwithdelims (){j-1}}, \end{aligned}$$
  with $0^{(k)}=0$. Eckhoff’s conjecture^59^, proven in^60^ holds that the *h*-numbers of a *d*-representable complex must satisfy the following inequalities:
  $$\begin{aligned} {\left\{ \begin{array}{ll} h_k\ge 0 &{} \text{ for } \,\,\, k=0,1,\ldots ; \\ h^{(k+1)}_k \le h_{k-1}, \,\,\, &{} \text{ for } \,\,\,k=1,2,\ldots ,D-1; \\ h^{(d)}_k\le h_{k-1}-h_k, \,\,\, &{} \text{ for } \,\,\, k=D,D+1,\ldots \,. \end{array}\right. } \end{aligned}$$
  which can be verified not only for “static” complexes, but also for the “inflating” (2) and “flickering” complexes, at each step of their evolution.
- *Qualitative spatial consistency*. It can be shown that if the RCC5 relationships among all triples of regions are consistent, then the entire schema ${\mathcal {R}}_5$ is consistent^93–99^. The full set of consistent triples is given in the following table.

- *Recognizing* 1*-representability* algorithm follows the exposition in^56,57^.

Let ${\mathcal {I}}=\{I_1=[a_1,b_1],\ldots ,I_n=[a_n,b_n]\}$ be set of intervals of a Euclidean line $R^1$.

##### Definition 1

$G({\mathcal {I}})$ is an interval graph, if each vertex $v_i\in G({\mathcal {I}})$ corresponds to an interval $I_i\in {\mathcal {I}}$ and a pair of vertexes $(v_i,v_j)$ is connected by an edge iff $I_i$ and $I_j$ intersect.

##### Definition 2

A *comparability graph* $G_{\lhd }$ represents an abstract relationship “$\lhd$”, if its vertexes $v_i$ represent elements of a set, and each link $e_{ij}$ represents a $\lhd$-related pair, $v_i \lhd v_j$.

**Figure 9:**
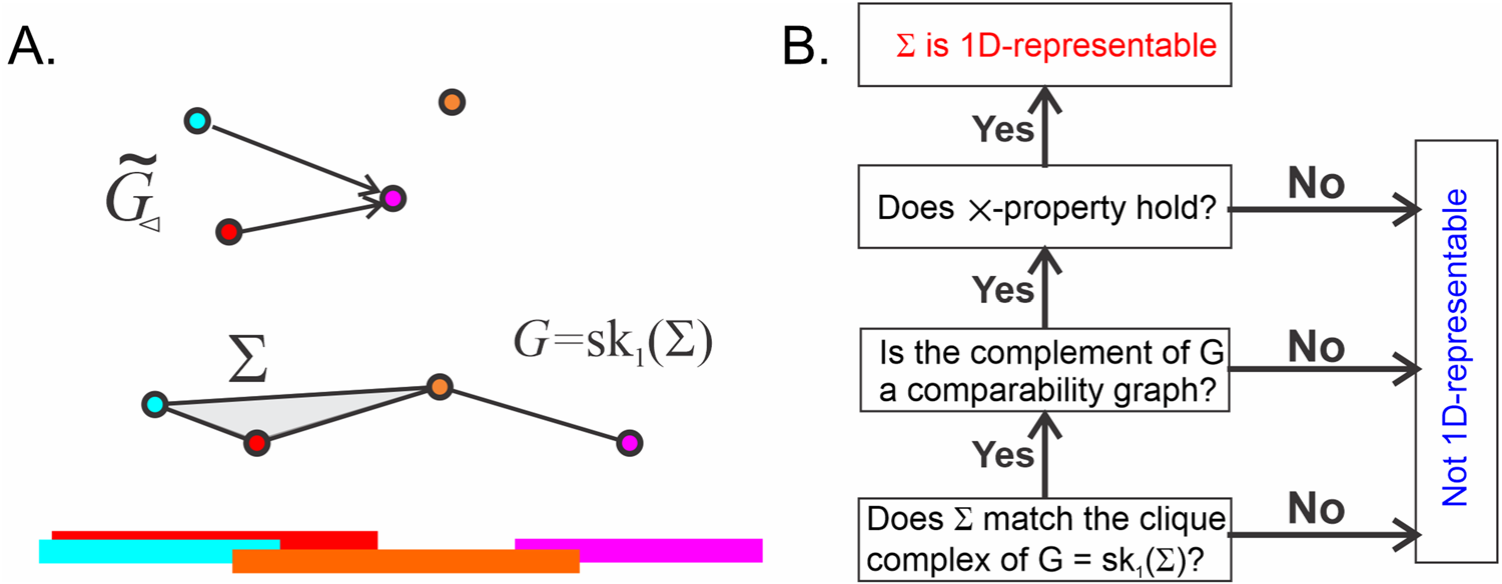
An algorithm for recognizing 1-representable complexes. (**A**) Four intervals covering a linear segment (bottom) can be represented by a simplicial complex—the nerve of the cover (middle panel). The vertexes of the corresponding interval graph $G({\mathcal {I}})$—the 1*D* skeleton of $\Sigma$—(color-coded) are connected if their respective intervals overlap, $I_i \cap I_j\implies v_i\lhd v_j$. The corresponding comparability graph, ${\tilde{G}}_{\lhd }({\mathcal {I}})$ is shown above, with the order indicated by arrows: $v_i \lhd v_j$ iff there is an arrow leading from $v_i$ to $v_j$. (**B**) Given a simplicial complex $\Sigma$, first check whether it is the clique complex of its 1*D*-skeleton $G:=sk_1(\Sigma )$. If it is not, then $\Sigma$ is not 1-representable; if it is, then check whether the complement graph of *G* is a comparability graph. If it is not, then $\Sigma$ is not 1-representable. If it is, then check the $\times$-property: if it holds, then $\Sigma$ is 1-representable, otherwise it is not.

An interval graph is hence 1-dimensional skeleton of the nerve of ${\mathcal {I}}$ (Fig. 9A). It can also be verified that the complement of an interval graph is a directed comparability graph ${\tilde{G}}_{\lhd } ({\mathcal {I}})$, in which the relationship $v_i\lhd v_j$ is defined by the order of the overlapping intervals,

9 $$\begin{aligned} v_i\lhd v_j \implies b_i<a_j. \end{aligned}$$

##### Definition 3

A directed graph satisfies $\Lambda$-property if there are no three vertices $v_i,v_j,v_k$ such that $v_i,v_k$ are not adjacent, while $v_i$ is adjacent to $v_j$ and $v_j$ is adjacent to $v_k$ with the corresponding orientations being $e_{ij}$ and $e_{jk}$ respectively.

##### Definition 4

A comparability graph satisfies $\times$-*property* if no four vertexes $v_i,v_j, v_k, v_l$ produce disjoint pairs of intervals.

In other words, a situation when $v_i\lhd v_j$ and $v_k\lhd v_l$ (i.e., the pair of intervals $(I_i,I_j)$ overlaps and the pair $(I_k,I_l)$ also overlaps), while the remaining pairs remain incompatible, e.g., $v_j \ntriangleleft v_k$, $v_j \ntriangleleft v_l$, (i.e., $I_j$ does not overlap either $I_k$ or $I_l$), etc., does not appear.

##### Theorem

A graph is an interval graph iff its complement is a comparability graph with an order defined by (9), satisfying the $\times$-property.

This theorem and the definitions motivate the following algorithm for identifying 1*D* representability of a complex $\Sigma$ (Fig. 9B):

1. Test whether $\Sigma$ is a clique complex, i.e., verify whether all $(k+1)-$tuples of vertexes $v_{\sigma }=[v_{i_0},v_{i_1},\ldots ,v_{i_k}]$ form a simplex in $\Sigma$ if and only if each pair of vertexes $[v_{i_p},v_{i_q}]\in v_{\sigma }$ is an edge in its 1-skeleton $G=sk_1(\Sigma )$. If at least one $v_{\sigma }$ fails this test, then $\Sigma$ is not a clique complex and hence not representable.
2. Build the complement ${\tilde{G}}$ of ${{\,\mathrm{sk}\,}}_1(\Sigma )$ and verify its comparability as follows:
  i. Choose an edge between $v_i$ and $v_j$ and define an orientation on it (e.g., $e_{ij}\ne e_{ji}$). If $e_{ij}$ was selected, then search for all vertexes $v_{j'}$ that are connected to $v_{j}$ but not to $v_{i}$ (Fig. 9A). If the edge between *j* and $j'$ is not yet oriented, select $e_{j'j}$. If it was already $(j'j)$-oriented, continue on; the opposite, $(jj')$-orientation implies that $\Sigma$ is not representable.
    If the orientation for new edges cannot be selected, pause the algorithm and dispose of all the edges that have already been oriented. Then pick another unoriented edge and restart the $\Lambda$-rule: keep applying it until the process comes to a halt and the next set of edges needs to be removed. Do this until all the edges are serviced and hence oriented.
  ii. Verify that no 3-tuple of vertexes $(v_i,v_j,v_k)$ forms an oriented 3-cycle. If such a cycle exists, $\Sigma$ is non-representable in 1*D*.
  iii. Verify that no triple of vertexes $(v_i,v_j,v_k)$ is “disconnected,” i.e., given $e_{ij}$ and $e_{jk}$, there must exist an edge between *i* and *k*. If any triple violates this condition, $\Sigma$ is not representable. Otherwise ${\tilde{G}}={{\,\mathrm{sk}\,}}_1(\Sigma )$ is a comparability graph with the order: $v_i\lhd v_j$ for each $e_{ij}$.
3. For every vertex $v_i$, compute the set of lesser points, $D(v_i)=\{v_j \in V: v_j\lhd v_i\}$. Then, for all pairs of vertexes $(v_i,v_j)$ check whether $D(v_i)$ is a subset of $D(v_j)$ or vice-versa. If at least one of these conditions is not satisfied, $\Sigma$ is not representable.

If this sequence of conditions is satisfied, $\Sigma$ is 1*D*-representable.
